# *Lavandula angustifolia* Essential Oil Inhibits the Ability of *Fusobacterium nucleatum* to Produce Volatile Sulfide Compounds, a Key Components in Oral Malodor

**DOI:** 10.3390/molecules29132982

**Published:** 2024-06-23

**Authors:** Ofir Rosner, Shiri Livne, Maria Bsharat, Shir Dviker, Uziel Jeffet, Shlomo Matalon, Nir Sterer

**Affiliations:** Department of Prosthodontics, Goldschleger School of Dental Medicine, Sackler Faculty of Medicine, Tel-Aviv University, Tel-Aviv 6997801, Israel; dr.livne@yahoo.com (S.L.); bsharatmaria@gmail.com (M.B.); shirdviker@gmail.com (S.D.); uzieljef@gmail.com (U.J.); matalons@post.tau.ac.il (S.M.); drsterer@gmail.com (N.S.)

**Keywords:** *Lavandula angustifolia*, *Fusobacterium nucleatum*, volatile sulfide compounds

## Abstract

Oral malodor still constitutes a major challenge worldwide. A strong effort is invested in eliminating volatile sulfur compound-producing oral bacteria through organic natural products such as essential oils. *Fusobacterium nucleatum* is a known volatile sulfur compound-producing bacteria that inspires oral malodor. The aim of the present study was to test the effect of lavender essential oil on the bacterium’s ability to produce volatile sulfide compounds, the principal components of oral malodor. Lavender (*Lavandula angustifolia*) essential oil was extracted by hydrodistillation and analyzed using GC-MS. The minimal inhibitory concentration (MIC) of lavender essential oil on *Fusobacterium nucleatum* was determined in a previous trial. *Fusobacterium nucleatum* was incubated anaerobically in the presence of sub-MIC, MIC, and above MIC concentrations of lavender essential oil, as well as saline and chlorhexidine as negative and positive controls, respectively. Following incubation, volatile sulfur compound levels were measured using GC (Oralchroma), and bacterial cell membrane damage was studied using fluorescence microscopy. Chemical analysis of lavender essential oil yielded five main components, with camphor being the most abundant, accounting for nearly one-third of the total lavender essential oil volume. The MIC (4 µL/mL) of lavender essential oil reduced volatile sulfur compound secretion at a statistically significant level compared to the control (saline). Furthermore, the level of volatile sulfur compound production attributed to 1 MIC of lavender essential oil was in the range of the positive control chlorhexidine with no significant difference. When examining bacterial membrane damage, 2 MIC of lavender essential oil (i.e., 8 µL/mL) demonstrated the same, showing antibacterial membrane damage values comparative to chlorhexidine. Since lavender essential oil was found to be highly effective in hindering volatile sulfur compound production by *Fusobacterium nucleatum* through the induction of bacterial cell membrane damage, the results suggest that lavender essential oil may be a suitable alternative to conventional chemical-based anti-malodor agents.

## 1. Introduction

Oral malodor is a common complaint among dental patients, affecting some 25% of the adult population [1,2]. It is estimated to be the third most frequent complaint of patients seeking dental care, following dental caries and periodontal disease [3]. This condition most often results from the production of malodorous metabolic by-products secreted by Gram-negative anaerobic oral bacteria [4]. The chemical compounds that formulate VSC are hydrogen sulfide, methyl mercaptan, and dimethyl sulfide [5]. The bacterium *Fusobacterium nucleatum* (Fn) has been implicated as a putative producer of volatile sulfide compounds (VSCs) that are considered major components in oral malodor manifestation [5]. It was proven to produce malodorous compounds depending on the amino acid to be disassembled [6,7,8,9].

Apart from being a periodontal pathogen, Fn has attracted attention since it has been associated with a broad spectrum of diseases, including head and neck infections, adverse pregnancy outcomes, inflammatory bowel disease, appendicitis, and colorectal cancers [7,8,9]. This obligately anaerobic thin rod-shaped Gram-negative bacterium of the oral cavity is known to act as a bridge between early and late colonizing bacteria in dental plaque. It has a crucial role in oral health biofilm formation, structure, ecology, and maturation [9,10,11]. It is located primarily on multispecies biofilms at the gingival margins adjacent to the tooth surface, where it manifests its’ periopathogenic features, but it may also be found on the dorsal surface of the tongue [12,13].

A large variety of dental products—mainly mouthwashes and dentifrices—containing a wide array of traditional chemically based antibacterial agents, including chlorhexidine, quaternary ammonium, chlorine dioxide, triclosan, and fluoride salts, has been suggested for the treatment of oral malodor with varying degrees of short-term success [14]. A previous study testing the effect of various herbal medicinals on malodor production in a salivary incubation assay demonstrated the ability of lavender to inhibit anaerobic bacterial growth and VSC production [15], most likely by inducing bacterial cell membrane damage [16].

During the last two decades, research on essential oil has evolved, and various beneficial effects on human health have been reported (antibacterial, antifungal, antioxidant, antiparasitic, antiseptic, antiviral, etc.) alongside insecticidal activities [17,18,19]. After more than a century of using antibiotic therapy, studies have shown an increase in resistance to antimicrobial drugs, both in Gram-negative and Gram-positive pathogens. Thus, some infections became untreatable, resulting in increased morbidity/mortality worldwide [20,21]. Moreover, the pathogens are increasingly resistant to common antibiotics, making the therapy management more difficult and impeding patients’ optimal treatment [20,21]. Consequently, the interest in the identification and development of natural alternative antibacterial agents is growing. A major subject of such extensive research is the Lavandula genus, originating from the Lamiaceae family. This genus covers some 30 species, but four of the species are more renowned for their popular use in cosmetics, perfume, and the pharmaceutical industry. One of the four derivatives is Lavandula angustifolia, also named English lavender or true lavender. Recently, several studies have reported on the therapeutic benefits of lavender essential oils (LEO), such as analgesic, antimutagenic, anti-inflammatory, and anxiolytic properties, as well as their extensive antimicrobial activities against a large number of microorganisms, including several Gram-negative bacteria [22,23,24,25,26]. Studies researching the impact of EOs on halitosis did not specifically include Fn [27], and studies researching the antibacterial activity of EOs on Fn did not focus specifically on LEOs [28]. To the best of our knowledge, this is the first study that tested the effect of LEO on the VSC production of FN, known as a key organism in oral malodor production. Therefore, we hypothesized that LEO may serve as a potential antimalodorous agent. The aims of this study include examining the effect of LEO on VSC production as well as ascertaining the mechanism of action by recording microorganism viability and cell membrane damage.

## 2. Results

### 2.1. Essential Oil Extraction and Chemical Analysis

The results of the GC-MS analysis are presented in Table 1. Sixty peaks were detected, with 22 compounds identified as main components with a concentration of more than 0.1%, out of which five compounds were most abundant, including eucalyptol, linalool, camphor, borneol, and linalyl acetate, accounting for more than 75% of the compounds.

### 2.2. Minimal Inhibitory Concentration (MIC) Determination

MIC was defined as the lowest concentration of lavender essential oil that inhibited the growth of the tested bacteria via no turbidity observed. The MIC value of lavender essential oil against F. nucleatum was 4 µL/mL.

### 2.3. Volatile Sulfide Compound (VSC) Determination

**Figure 1:**
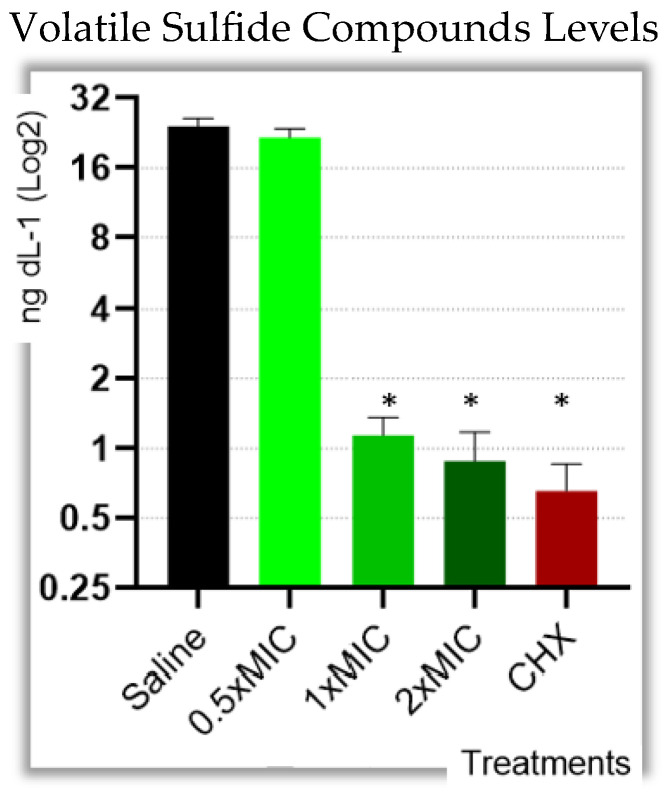
Effect of various concentrations of lavender essential oil on volatile sulphur compounds produced by F. nucleatum determined by using Oralchroma and expressed in ng/mL. Results are presented in mean ± standard deviation. Asterisk denotes differences with significant value (*p* < 0.05).

Volatile sulfur compound levels were significantly reduced when F. nucleatum was exposed to lavender essential oil at concentrations of 4 μL/mL and above (*p* < 0.001). The effect of lavender essential oil against the production of VSCs is summarized in Figure 1.

### 2.4. Bacterial Cell Membrane Integrity Determination

**Figure 2:**
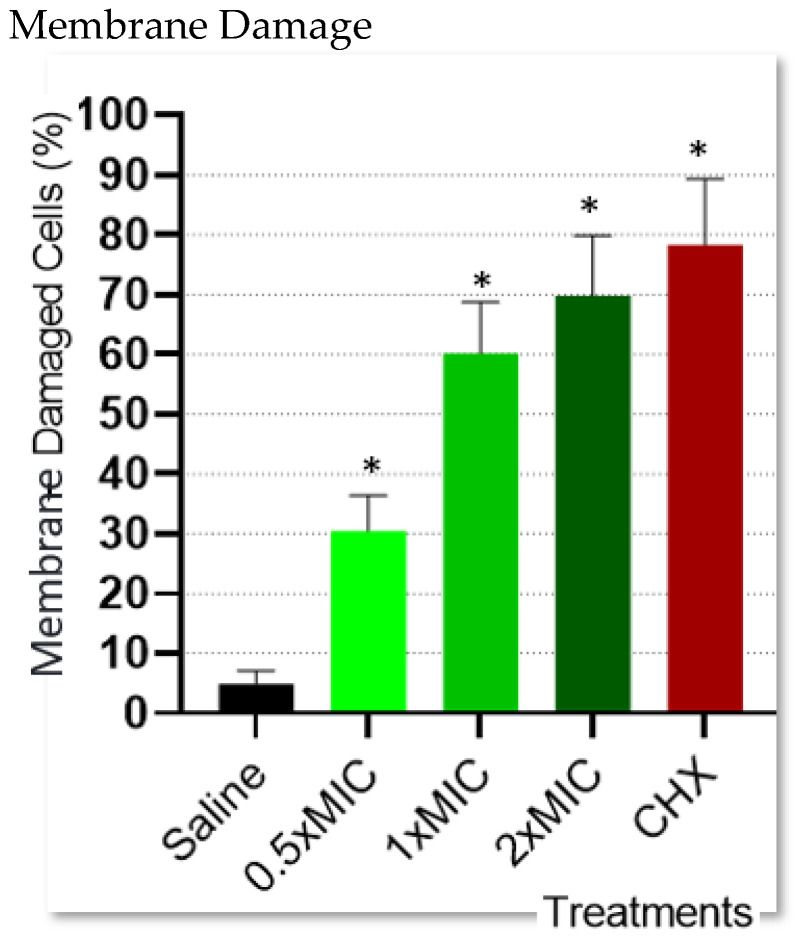
The effect of various concentrations of lavender essential oil on bacterial cell membrane integrity determined by live/dead staining via fluorescent microscopy and expressed in percentage of live and dead bacteria. Results are presented in mean ± standard deviation. Asterisk denotes differences with significant value (*p* < 0.05).

**Figure 3:**
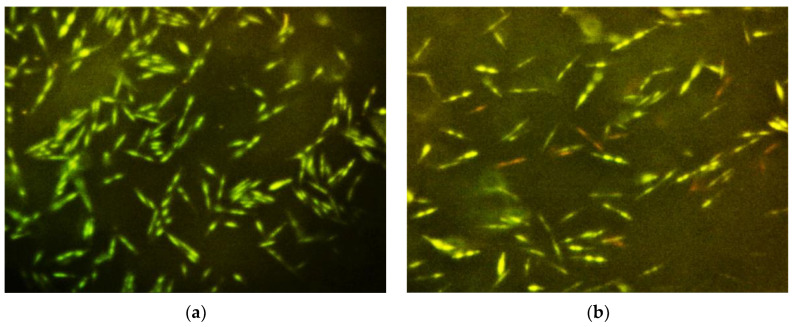

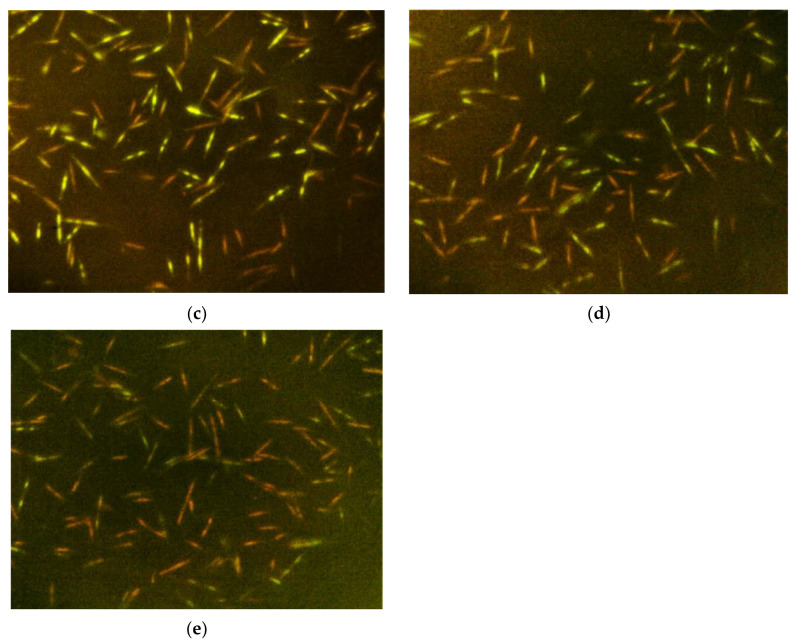
Fluorochrome microscopic images of F. nucleatum exposed to various concentrations of lavender essential oil and 0.2% chlorhexidine solution (green: live cell; red: dead cell). (**a**) Saline; (**b**) 0.5 × MIC lavender essential oil; (**c**) MIC lavender essential oil; (**d**) 2 × MIC lavender essential oil; (**e**) 0.2% chlorhexidine.

After exposure to lavender essential oil at the MIC or 2 × MIC, the integrity of the bacterial cell membrane was destroyed by approximately 60% to 70%. Less than 50% of bacterial cell membrane integrity was disrupted by the low concentration at 0.5 × MIC and saline. The effect of various concentrations of lavender essential oil on bacterial cell membrane integrity is shown in Figure 2 and Figure 3.

A visual depiction of the antibacterial effect made by LEO is illustrated through fluorescence microscopy images (Figure 3). Assessing the pictures visually gives the impression of a gradual cell membrane destruction. There was no visible cell membrane destruction when exposed to saline (the negative control (Figure 3a)) compared to immense cell membrane destruction, undistinguishable between 2 MIC LEO and 0.2% CHX—the positive control (Figure 3d and Figure 3e, respectively).

## 3. Discussion

Lavender oil has been reported as an antibacterial agent that might be a viable alternative to conventional antimicrobial therapy. An in vitro study on the antibacterial activity of LEO against antibiotic-resistant bacteria demonstrated its bactericidal effect [29].

The results of the present study displayed minimal inhibitory concentration (MIC) values of LEO against *Fusobacterium nucleatum,* showing effectiveness in reducing VSC production. These results also showed that LEO induced cell membrane damage in 30% of bacterial cells in concentrations as low as half of the MIC levels, suggesting an explanation for the underlying mechanism for the VSC reduction. As mentioned earlier, the importance of *Fusobacterium nucleatum* is twofold: acting as a VSC-producing bacteria and serving as a bridge to late biofilm colonizers who act as known VSC-producing bacteria [6]. Furthermore, those late colonizers are also involved in periodontal disease progression, a part of their malodorous role [6]. LEOs were documented to possess antibacterial effects against those microorganisms, such as *P. Gingivalis* [30,31] and *P. Intermedia* [30,31]. Camphor, one of the most abundant ingredients detected in the LEO chemical analysis, is also known for its antibacterial activity against *P. Gingivalis* and *P. Intermedia* [32]. This strategic importance made *Fusobacterium nucleatum* the primary target bacterium in our study. The premise that the antibacterial effect (through bacterial cell damage) might be the underlying mechanism for the VSC reduction is in accordance with another study that reported tea extract effects on *Fusobacterium nucleatum,* demonstrating both increased cell membrane permeability concomitantly with a reduction in VSC production [33].

A previous in vivo study showed a clinical relationship between herbal extracts, including *Lavandula angustifolia*, and a significant reduction in VSC production. This effect was superior to zinc and chlorhexidine, yet no microbial mechanism has been suggested to explain such results [34]. Our in vitro study strives to shed light on that topic, elucidating the antibacterial mechanism shown here, specifically on Fn.

In parallel literary reports, *Fusobacterium nucleatum* was exposed to additional EOs and not just LEOs. Labrador tea, peppermint, and winter savory EOs were all immersed with three strains of that target bacteria. MIC values with the first two EOs were within the range of MIC values reported in our research (2.5–5 µL/cc). Winter savory EO demonstrated even one magnitude better bacteriostatic efficiency than in our report, with an MIC value of 0.3–0.6 µL/cc [35]. It should be mentioned that none of the five compounds our chemical analysis revealed were as abundant as any of the EOs mentioned above.

Chemical analysis of the extracted oil used in this study demonstrated the presence of 22 main compounds, all of which are known from the current literature regarding the hydrodistillation of lavender oil [36,37]. Five of these compounds, eucalyptol, linalool, camphor, borneol, and linalyl acetate, were highly abundant in the oil, accounting for over 75% of the total volume. Camphor was most abundant, showing a concentration that was twice as high as the standard range reported by the European pharmacopeia [35,36,37]. As expected, almost all of the compounds registered were terpenes and their derivatives, as well as terpenoids and their derivatives. Monoterpenoids and terpenoids account additively for more than 70% of the total volume. Apart from L-α-Terpineol, Terpinen-4-ol, and Lavandulyl acetate, none of the listed constituents identified in our analysis met the registered criteria defined in the European Pharmacopeia (10th Ed) for LEO [38]. Other researchers also reported that their Lavender cultivars did not conform with the official standard defining LEO according to the European Pharmacopeia [39,40,41].

A recent study reported that combining lavender oil with camphor in its powdered form has synergistically increased the antibacterial properties of these substances [42]. This is in agreement with our findings, showing high camphor concentrations in the chemical composition of LEO used in the present study.

Various essential oils extracted from different sources, such as white cypress oil, cinnamon oil, coriander oil, lemongrass oil, clove oil, eucalyptus oil, Juniper oil, and Peppermint oil, share common compounds also found in LEO (e.g., camphor, eucalyptol) that have exhibited an antibacterial effect against *Fusobacterium nucleatum* [32]. Another study that demonstrated the antibacterial effect of LEO against various types of microorganisms, including Gram-positive bacteria, Gram-negative bacteria, and fungi, conducted on LEO extracted from the species *Lavandula stoechas* also recorded elevated levels of camphor (i.e., 28%) [43]. Furthermore, a second study measuring the antibacterial effect of *Lavandula stoechas* against periopathogenic bacteria, including two strains of *Fusobacterium nucleatum* (ATCC 25586 and AHN 9508), reported very high levels of camphor (49%) and an MIC value of 4 µL/mL, similar to our findings [30].

Another abundant chemical constituent detected in the analysis was linalool, accounting, with its derivatives, for 18–19% of the total volume. Some authors even consider that the activity of linalool reflects that of the whole oil, indicating its dominant activity in LEO [44]. It is known to have an antibacterial effect against bacteria and fungi [45], some of which are oral bacteria, both cariogenic and periopathogenic. Park and his co-workers demonstrated MIC values of linalool within the range of our research, with five subspecies of *Fusobacterium nucleatum* besides the cariogenic bacteria [46]. Together with camphor, comprising additively with linalool more than half of the total oil volume in this study, there is little surprise that LEO proved to be such a potent bacteriostatic EO.

In addition to the promise essential oils hold in vitro, there are still several challenges involved in their clinical application, such as poor oral bioavailability and sensitivity to oxygen and humidity [47]. However, the development of suitable delivery systems that may provide ways to overcome these issues, such as the incorporation of LEO into different types of nanoparticle delivery systems that may provide a more sustained and controlled release, has been suggested [32].

Within the limitation of an in vitro study, taken together, the results of the present study suggest that LEO may serve as a potent antimalodorous agent against the production of VSC by *Fusobacterium nucleatum* and that this effect is mediated through cell membrane damage and may be attributed with high levels of camphor and linalool.

## 4. Materials and Methods

### 4.1. Essential Oil Extraction and Chemical Analysis

Dried flowers of *Lavandula angustifolia* were subjected to hydrodistillation for four hours. Dried flowers were kindly provided by Dr. Yossi Rubinstein (Agrimed, Weizmann Institute, Ness Ziona) [15]. All methods were carried out in accordance with relevant guidelines. The extracted essential oil (0.4 mL) was dried using lyophilizer. The dried sample was dissolved in 0.5 mL of acetonitrile and injected into a model 7890B gas chromatograph coupled with a model 5977A Mass Selective Detector (Agilent Technologies Inc., Santa Clara, CA, USA). The analytical column was a fused-silica capillary DB5-MS UI (30 m × 0.25 mm ID × 0.25 μm film thickness) (PN: 122-5532UI, Agilent Technologies Inc., Santa Clara, CA, USA). The oven temperature program started at 40 °C for 3 min before ramping at a rate of 3 °C min^−1^ to 250 °C and 20 °C min^−1^ to 325 °C for 0.25 min. Helium (99.99% purity) was used as a carrier gas with a flow rate of 1.2 mL min^−1^. The injector and detector temperatures were 250 °C and 280 °C, respectively. Sample was injected using a split ratio of 24:1. MS was set at detection gain of 1.0, and the scan was conducted from 45 to 600 amu.

### 4.2. Minimal Inhibitory Concentration (MIC) Determination

*Fusobacterium nucleatum* (PK1594) was cultured in brain heart infusion (BHI) broth for 48 h at 37 °C under anaerobic conditions using an anaerobic jar and kit (GasPack^®^ EZ, BD, Pontypridd, UK) as reported in a previous experiment [16].

In a preliminary experiment, two-fold dilutions of *Lavandula angustifolia* essential oil in BHI broth were prepared in a 96-well microplate (with concentrations ranging from 128 to 0.125 mL/mL). Wells were inoculated with 10 mL of *Fusobacterium nucleatum* suspension adjusted to a density of 0.4 McFarland standard (equivalent to 10^6^ CFU/mL). Microplates were incubated for 48 h at 37 °C under anaerobic conditions. MIC was determined as the minimal concentration in which no bacterial growth was observed.

### 4.3. Volatile Sulfide Compound (VSC) Determination

Volatile sulfide compound (VSC) levels were measured in the test tubes using a portable gas chromatograph (OralChromaTM, Abilit Corp., Osaka, Japan). Headspace samples (0.5 mL) were collected from the test tubes using disposable syringes and injected into the inlet valve of the measuring device. Results were recorded in ng/dL of total VSC levels [16].

### 4.4. Bacterial Cell Membrane Integrity Determination

Test tubes containing 3 mL of BHI broth were added with Lavandula angustifolia essential oil at a final concentration of 2, 4, and 8 µL/mL (equivalent to 0.5 × MIC, 1 × MIC, and 2 × MIC, respectively) as well as Saline and 0.2% chlorhexidine as negative and positive controls, respectively. Test tubes were inoculated with 100 µL of Fusobacterium nucleatum suspension (0.4 OD, 600 nm) and incubated for 48 h at 37 °C under anaerobic conditions. Following incubation, these suspensions were analyzed for volatile sulfide compound levels and bacterial cell membrane damage, as described below.

Membrane damage was determined using a Bacteria Live/Dead Staining Kit^®^ (Promokine, Heidelberg, Germany), which comprised DMAO, a green-fluorescent dye that binds nucleic acids of both membranes intact and membrane-damaged bacterial cells, and Ethidium homodimer-III (EthD-III), a red-fluorescent dye that binds only to nucleic acids of membrane-damaged bacterial cells. Bacterial samples (100 µL) were added with 1 µL of the dye mixture and incubated in the dark for 15 min at room temperature. The samples (10 µL) were wet mounted on slides, covered with cover slips, and studied under a fluorescent microscope (×1000, L3201LED, MRC), using an excitation light of 460–470 nm and a blue LED filter with cutoff of 500 nm. Digital images of six random fields were taken using a mounted camera (AM7023, DinoEye^®^, Anmo, Taiwan, China). Results were recorded as percentage of red fluorescent dyed bacteria of total counts.

### 4.5. Statistical Analysis

To compare the effect of the various treatments on the parameters tested, ANOVA was applied with post-hoc pairwise comparisons according to Dunnet and Scheffe. Tests applied were two-tailed, and *p* ≤ 0.05 was considered statistically significant. Experiments were conducted in six replicates. The parameters tested were total VSC production and cell membrane damage.

## Figures and Tables

**Table 1:** Identification of the main components found in the *Lavandula* essential oil.

Compound	Retention Time (min)	Quantification ^1^
Camphene	10.911	0.72
p-Cymene	14.57	0.22
Eucalyptol	14.955	17.06
Lavender lactone	15.094	0.41
Cis-Linalool oxide (furanoid)	16.898	6.5
trans-Linalool oxide (furanoid)	17.68	4.5
Linalool	18.444	7.72
Octen-3-yl-acetate	18.841	0.35
Camphor	20.601	33.26
Borneol	21.756	7.21
Trimethyl-6-vinyltetrahydro-2H-pyran-3-ol	21.945	0.42
Terpinen-4-ol	22.147	0.19
Para-cymen-8-ol	22.481	0.11
α-Terpineol	22.866	0.57
Linalyl acetate	25.592	12.18
Bornyl acetate	26.998	0.3
Lavandulyl acetate	27.106	1.43
Neryl acetate	30.399	0.1
Geranyl acetate	31.257	0.24
α-santalene	32.853	0.55
Caryophyllene oxide	39.288	1.42

^1^ % of total.

## Data Availability

Data is contained within the article.
